# Unique Phenotypes of Heart Resident Type 2 Innate Lymphoid Cells

**DOI:** 10.3389/fimmu.2020.00802

**Published:** 2020-05-05

**Authors:** Yafei Deng, Shuting Wu, Yao Yang, Meng Meng, Xin Chen, Sha Chen, Liping Li, Yuan Gao, Yue Cai, Saber Imani, Bingbo Chen, Shuhui Li, Youcai Deng, Xiaohui Li

**Affiliations:** ^1^Institute of Materia Medica, College of Pharmacy, Army Medical University (Third Military Medical University), Chongqing, China; ^2^Hunan Children’s Research Institute (HCRI), Hunan Children’s Hospital, Changsha, China; ^3^Department of Clinical Biochemistry, Faculty of Pharmacy and Laboratory Medicine, Army Medical University (Third Military Medical University), Chongqing, China; ^4^Southwest Hospital/Southwest Eye Hospital, Army Medical University (Third Military Medical University), Chongqing, China; ^5^Department of Cardiology, Xijing Hospital, Fourth Military Medical University, Xi’an, China; ^6^Department of Oncology, The Affiliated Hospital of Southwest Medical University, Luzhou, China; ^7^Laboratory Animal Center, Army Medical University (Third Military Medical University), Chongqing, China

**Keywords:** innate lymphoid cells, heart, ILC2s, IL-4, IL-33

## Abstract

Innate lymphoid cells (ILCs), including ILC1s, ILC2s, and ILC3s, play critical roles in regulating immunity, inflammation, and tissue homeostasis. However, limited attention is focused on the unique phenotype of ILCs in the heart tissue. In this study, we analyzed the ILC subsets in the heart by flow cytometry and found that ILC2s were the dominant population of ILCs, while a lower proportion of type 1 ILCs (including ILC1 and NK cells) and merely no ILC3s in the heart tissue of mice. Our results show that ILC2 development kinetically peaked in heart ILC2s at the age of 4 weeks after birth and later than lung ILC2s. By conducting parabiosis experiment, we show that heart ILC2s are tissue resident cells and minimally replaced by circulating cells. Notably, heart ILC2s have unique phenotypes, such as lower expression of ICOS, CD25 (IL-2Rα), and Ki-67, higher expression of Sca-1 and GATA3, and stronger ability to produce IL-4 and IL-13. In doxorubicin-induced myocardial necroptosis model of mouse heart tissue, *IL-33* mRNA expression level and ILC2s were remarkably increased. In addition, IL-4 production by heart ILC2s, but not lung ILC2s, was also dramatically increased after doxorubicin treatment. Our results demonstrate that heart-resident ILC2s showed tissue-specific phenotypes and rapidly responded to heart injury. Thus, further studies are warranted to explore the potential for IL-33-elicited ILC2s response as therapeutics for attenuating heart damage.

## Introduction

Innate lymphoid cells (ILCs), which are widely distributed in the body and lack the type of diversified antigen receptors, are the innate counterparts of T lymphocytes (1, 2). It is well accepted that ILCs are identified as lineage-negative (Lin^–^) and interleukin-7 (IL-7) receptor α-positive (CD127^+^) (3), emerging into three populations (ILC1s, ILC2s, and ILC3s) based on the signature transcription factors and effector cytokines. ILC1s require the transcription factor T-bet and produce interferon-gamma (IFN-γ), ILC2s express the transcription factor GATA3 and produce the type 2 cytokines IL-4, IL-5, and IL-13, while ILC3s express the transcription factor RAR-related orphan receptor gamma t (RORγt) and have the ability to produce IL-22 and/or IL-17 (4, 5).

Growing evidence suggest that ILC subsets are involved in development of specific tissue tropisms, including the skin, intestine, liver and lung (1). For example, ILC1s are the dominant ILC population in intestinal intraepithelial layer (IEL) and liver, whereas ILC2s are the dominant population in the lung and skin. ILC3s are found in significant numbers in intestinal lamina propria layer (6–10). To date, the ILC subsets are poorly characterized in tissue homeostasis and tissue-specific response after injury in heart tissue. Most recently, a group of non-cytotoxic cardiac ILC progenitor was found in the heart tissue, suggesting that ILCs with specific-feature may also exist (11).

Here, we found that ILC2s are the dominant population of ILCs, while ILC1s are also present with a lower proportion and there are no ILC3s in the mice heart tissue. Compared with lung ILC2s, heart ILC2s have unique phenotypes in the identified markers and the ability of IL-13 and IL-4 cytokines secretion. Furthermore, ILC2s rapidly expanded and secreted IL-4 in response to myocardial necroptosis.

## Materials and Methods

### Animals

Male or female C57BL/6 mice [vary from embryonic day (E) 18.5-8 weeks old] were maintained under specific pathogen free conditions, which were acclimatized at 22–25°C, 50 ± 10% relative humidity and had 12 h light/dark cycles, periodic air changes, and free access to water and food in the Experimental Animal Center of the Army Military Medical University (Chongqing, China). Congenic C57BL/6 CD45.1 mice strains were obtained from The Jackson Laboratory (Sacramento, CA, United States). All animal procedures and protocols were approved by the Animal Ethics Committee of the Army Medical University, and followed the guidelines of the Institutional Animal Care and Use Committees of the Army Military Medical University (Chongqing, China).

### Parabiosis

Parabiosis were performed as previously described in the literature (12, 13). Briefly, mice were anesthetized by isoflurane vaporizer (4–5% v/v). Then skin incisions were made on the flanks of age-, sex- and weight-matched CD45.2^+^ (C57BL/6), besides CD45.1^+^ (C57BL/6) mice followed by gently detaching the skin from the subcutaneous fascia. The knee joints of two mice are clearly distinguishable, connected and then the incisions were joined with a continuous absorbable suture. 0.5 ml of 0.9% NaCl was administrated subcutaneously to each mouse to prevent dehydration and post-operatively. Mice received pain medication and antibiotics for the first week after parabiosis.

### Doxorubicin (DOX)-Induced Myocardial Necroptosis

Eight weeks old C57BL/6 mice were injected with either DOX (20 mg/kg, i.p., Med Chem Express LLC, Shanghai, China) or saline, according to a previous study (14). Heart tissues were collected and single-cell suspensions were prepared by enzymatic digestion after 24 h or 96 h of DOX treatment.

### Single-Cell Suspensions Preparation

Liver tissues were grinded and passed through a 70-μm stainless steel mesh. Then, cells were resuspended in 35% Percoll (GE Healthcare, Pittsburgh, PA, United States) and pellets were collected after centrifugation (450 × *g*, room temperature, 10 min). The liver mono-nuclear cells were separated from the pellets through lysing erythrocytes (15). For heart and lung lymphocyte isolation, the fresh mouse heart was perfused with cold PBS to remove peripheral blood cells. Briefly, mice were anesthetized by isoflurane vaporizer (4–5% v/v). The heart was slowly perfused with cold PBS from left ventricle by a 10 ml-syringe until the fluid was clear. Then heart and lung tissues were cut into pieces and then digested for 45 min at 37°C in Hank’s solution containing 10% FBS and 1 mg/ml collagenase I (Sigma-Aldrich, St Louis, MO, United States), 1 mg/ml collagenase II (Gibco, Waltham, MA, United States) and 25 μg/ml DNase I (Sigma-Aldrich, St Louis, MO, United States). After digestion, the cells were then resuspended in 20% percoll in PBS (pH 7.4, Sigma-Aldrich, St Louis, MO, United States) and pellets were collected after centrifugation (450 × *g*, room temperature, 10 min) (16). For small intestines lamina propria layer lymphocyte isolation, luminal contents were flushed and peyer’s patches were removed. Then the intestines were opened lengthwise and gently agitated for 20 min at 37°C in D-hank’s solution (pH 7.4) containing 10 mM HEPES, 5 mM EDTA and 1 mM DTT. Tissues were then rinsed with Hank’s solution prior to digestion with 1 mg/ml collagenase II for 40 min at 37°C under agitation. The collected digests were filtered through 100 micron mesh and subjected to centrifugation (450 × *g*, room temperature, 10 min) using 25% percoll solutions (17).

### Antibodies and Flow Cytometry

Antibodies used for flow cytometry were commercially purchased and are listed in Table 1. We confirmed the species reactivity for all antibodies according to the official directions and performed preliminary experiments to determine the appropriate dilution for all antibodies. Standard protocols were followed for flow cytometry (18, 19). Briefly, single-cell suspensions were obtained from the heart, lung, liver and intestinal lamina propria tissue of mice. For surface markers, 2 × 10^6^ cells were stained with anti-CD16/CD32 antibodies (eBioscience, San Diego, CA, United States) 15 min at room temperature, in the dark with staining buffer (phosphate-bufferd saline (PBS) containing 2% mouse serum, 2% horse serum, and anti-CD16/CD32 blocking antibodies). For intracellular IL-4, IL-5, and IL-13 staining, 2 × 10^6^ cells were stimulated with IL-33 (eBioscience, San Diego, CA, United States) or PMA/ionomycin (BD Biosciences, San Diego, CA, United States) plus BD Golgi Plug protein transport inhibitor (BD Biosciences, San Diego, CA, United States) for 4 h, then cells were fixed with Fixation/Permeabilization Solution Kit (BD Biosciences, San Diego, CA, United States) following the manufacturer’s instructions. RORγt, GATA3 and Ki67 were stained as recommended by the manufacturer using Foxp3/Transcription Factor Staining Buffer Set Kit (eBioscience, San Diego, CA, United States). Lineage (Lin) markers included CD3e, CD19, B220 and Gr-1. Isotype-matched control antibodies were all purchased from Biolegend (Biolegend, San Diego, CA, United States) and BD (BD bioscience, CA, United States) and used at the same concentration as test antibodies. All flow cytometry experiments were carried out on a BD FACS Verse or BD FACS Canto (BD Biosciences, San Diego, CA, United States); 500,000 – 1,000,000 events were assessed per condition within 1 h. Data were analyzed with FlowJo software (version 10.0, FlowJo LLC, Ashland, OR, United States). The lines indicate median values for each group.

**TABLE 1:** Antibodies used for flow cytometry.

**Antibodies**	**Clone**	**Source**	**Dilution**
Anti-mouse CD45	30-F11	BioLegend	1/200
Anti-mouse CD3e	145-2C11	BioLegend	1/200
Anti-mouse CD19	6D5	BioLegend	1/200
Anti-mouse B220	RA3-6B2	BioLegend	1/200
Anti-mouse Gr-1	RB6-8C5	BioLegend	1/200
Anti-mouse CD127	A7R34	BioLegend	1/100
Anti-mouse CD90.2	30-H12	BioLegend	1/100
Anti-mouse NK1.1	PK136	BioLegend	1/100
Anti-mouse NKp46	29A1.4	BioLegend	1/100
Anti-mouse CD49b	DX5	BioLegend	1/100
Anti-mouse KLRG1	2F1	BioLegend	1/100
Anti-mouse GATA3	16E10A23	BioLegend	1/20
Anti-mouse ICOS	15F9	BioLegend	1/100
Anti-mouse Sca-1	D7	BioLegend	1/100
Anti-mouse CD25	3C7	BioLegend	1/100
Anti-mouse F4/80	BM8	BioLegend	1/100
Anti-mouse CD11b	M1/70	BioLegend	1/100
Anti-mouse CD11c	N418	BioLegend	1/100
Anti-mouse MHC II	M5/114.15.2	BioLegend	1/100
Anti-mouse IL-4	11B11	BioLegend	1/50
Anti-mouse IgG2a	RTK2758	BioLegend	1/100
Anti-mouse IgG2b	RTK4530	BioLegend	1/100
Anti-mouse IgG	SHG-1	BioLegend	1/100
Anti-mouse IgG2a	RTK2758	BioLegend	1/100
Anti-mouse IgG2b	MPC-11	BioLegend	1/100
Anti-mouse CD49a	Ha31/8	BD Biosciences	1/100
Anti-mouse ST2	U29-93	BD Biosciences	1/100
Anti-mouse RORγt	Q31-378	BD Biosciences	1/100
Anti-mouse CD16/CD32	2.4G2	BD Biosciences	1/100
Anti-mouse CD4	RM4-5	BD Biosciences	1/100
Anti-mouse CD8a	53-6.7	BD Biosciences	1/100
Anti-mouse Ki-67	B56	BD Biosciences	1/66
Anti-mouse CD45.1	A20	BD Biosciences	1/100
Anti-mouse CD45.2	104	BD Biosciences	1/100
Anti-mouse IL-13	eBio13A	eBioscience	1/50
Anti-mouse IL-5	TRFK5	eBioscience	1/50

### Histological Analysis

Histological structures of heart were determined by standard hematoxylin-eosin (HE) staining. Briefly, resected specimens were fixed in 10% neutral buffered formalin for at least 24 h, embedded in paraffin, and 4 μm-thick sections were cut. After processing the sections according to standard protocols, they were stained with hematoxylin and eosin. The coverslips were visualized under a Leica confocal laser-scanning microscope (Leica, Wetzlar, Germany). The investigators were blinded for acquiring the images.

### RNA Isolation and qRT-PCR Analysis

To quantify the expression of mRNA, qRT-PCR was performed according to standard protocols as previously described (20). Total RNA was extracted from heart tissue using Trizol (Invitrogen, Waltham, MA, United States) and total RNA (1 μg) was then reverse-transcribed into cDNA using a First Stand cDNA Synthesis Kit (DBI Bioscience, Ludwigshafen, Germany). Real-time PCR reactions were carried out with Bestar SYBR Green qPCR master mix (DBI Bioscience, San Diego, CA, United States) using an ABI Prism 7700 Sequence Detector. The cycle threshold (Ct) values were normalized by the internal control β-actin. Primer sequences for qRT-PCR, obtained from reported literatures or designed by Pubmed Primer-BLAST. The primer pairs used were as follows: *IL-33* forward, 5′- CCCTGGTCCCGCCTTGCAAAA-3′; *IL-33* reverse, 3′- AGTTCTCTTCATGCTTGGTACCCGA-5′; *IL-25* forward, 5′-ACAGGGACTTGAATCGGGTC-3′; *IL-25* reverse, 3’- TGGTAAAGTGGGACGGAGTTG-5′; β*-actin* forward, 5′- GCCAACCGTGAAAAGATGAC-3′; and β*-actin* reverse, 3′-CATCACAATGCCTGTGGTAC -5′ (21).

### Statistical Analysis

All quantitative data were transferred to Excel and the statistical analyses were computed with SPSS software for Windows (Version 21, SPSS Inc., Chicago, IL, United States). Data are expressed as means ± S.E.M. For comparison between two independent experimental groups, an unpaired two-tailed Student’s *t*-test when data were normally distributed. When three or more independent groups were compared, one-way ANOVA followed by Tukey’s test was performed. A *p-*value less than 0.05 was considered to be statistically significant. In each analysis, there were *n* = 3–11 replicates per group and results were representative of at least two independent experiments. Sample size for each experiment is described in the corresponding figure legend. All graphs were produced by GraphPad Prism 5.0 for windows software (GraphPad Software Inc., La Jolla, CA, United States).

## Results

### ILC2s Are the Predominant Subset Among ILCs in Mouse Heart Tissue

**FIGURE 1:**
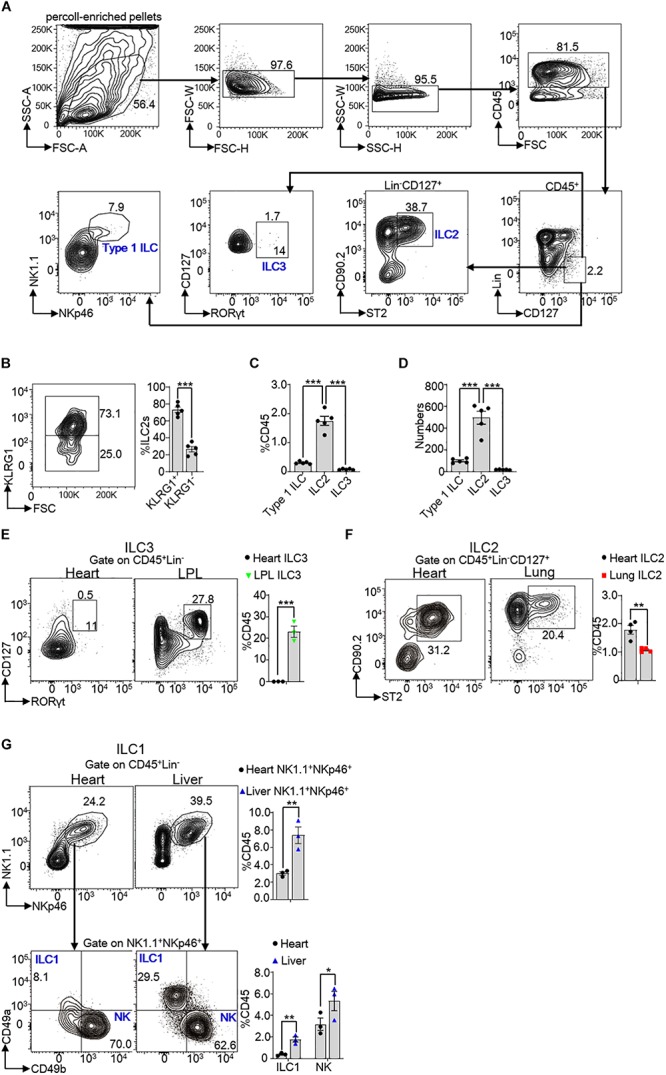
Subsets of ILCs in mouse heart tissue. **(A)** Gate strategy of ILCs in the heart of mice. Lineage (Lin) markers included CD3e, CD19, B220, and Gr-1. The number inside of gate indicates cell events. **(B)** Expression of KLRG1 in heart ILC2s of 8 weeks old mice. **(C,D)** Cumulative frequencies **(C)** and enumeration **(D)** of Type I ILCs (including ILC1s and NK cells), ILC2s and ILC3s in CD45^+^ lymphocyte in the heart of 8 weeks old mice. **(E)** Cumulative frequencies of ILC3s in CD45^+^ lymphocyte in heart and LPL of 8 weeks old mice. The number inside of gate indicates cell events. **(F)** Cumulative frequencies of ILC2s among CD45^+^ lymphocyte in the heart and lung tissue of 8 weeks old mice. **(G)** Another gate strategy of ILC1s irrespective of CD127 expression and cumulative frequencies of ILC1s (CD45^+^Lin^–^NK1.1^+^NKp46^+^CD49a^+^CD49b^–^) and NK cells (CD45^+^Lin^–^NK1.1^+^NKp46^+^CD49a^–^CD49b^+^) in heart and liver of 8 weeks old mice. Each dot represents one mouse; error bars represent SEM; *p < 0.05, **p < 0.01, ***p < 0.001. Unpaired two-tailed Student’s *t*-test **(B,E–G)**. One-way ANOVA **(C,D)**.

In order to investigate the subsets of ILCs in heart tissue, we collected heart lymphocyte mixture by lymphocyte separation from 8 weeks old mouse heart. Percoll-enriched pellets were resuspended and stained with surface and/or intracellular antibodies. Gate strategy of heart ILC subsets was shown in Figure 1A. We identified a population of lineage negative (Lin^–^) and CD127 positive cells in the CD45^+^ cells. Type I ILCs were identified by CD45^+^Lin^–^CD127^+^NK1.1^+^NKp46^+^ (including ILC1 and NK cells), ILC2s were identified by CD45^+^Lin^–^CD127^+^CD90.2^+^ST2^+^ and ILC3s were recognized by CD45^+^Lin^–^CD127^+^RORγt^+^ (8, 15, 22). We found that ILC2s were divided into KLRG1^+^ ILC2s and KLRG1^–^ ILC2s (Figure 1B). Among CD45^+^cells, Type I ILCs accounted for about 0.2% (∼100 cells/per heart) and ILC2s accounted for about 1.7% (∼500 cells/per heart) (Figures 1C,D). Whereas, there were merely no ILC3s (∼18 cells/per heart) based on gate strategy used in the intestinal LPL ILC3s (Figure 1E). The ratios of ILC2s among CD45^+^ cells were higher in the heart tissue in compared with lung ILC2s of 8 weeks old mice (∼1.7-fold) (Figure 1F). As some studies reported that some ILC1 subsets, such as liver ILC1s and salivary ILC1s (23, 24), did not express CD127, we also used CD45^+^Lin^–^NK1.1^+^NKp46^+^CD49a^+^CD49b^–^ to gate ILC1s. and CD45^+^Lin^–^NK1.1^+^NKp46^+^CD49a^+^CD49b^–^ ILC1s accounted for about 0.4% of CD45^+^cells, which suggested that part of ILC1s also did not express CD127 in murine heart tissue. Besides, conventional NK cells accounted for about 3.0% of CD45^+^cells in the mouse heart (Figure 1G). Together, these data demonstrated that ILC2s were the most predominant subset of ILCs in mouse heart tissue, even greater than in lung tissue.

### Heart ILC2s Peak at the Age of 4 Weeks After Birth

**FIGURE 2:**
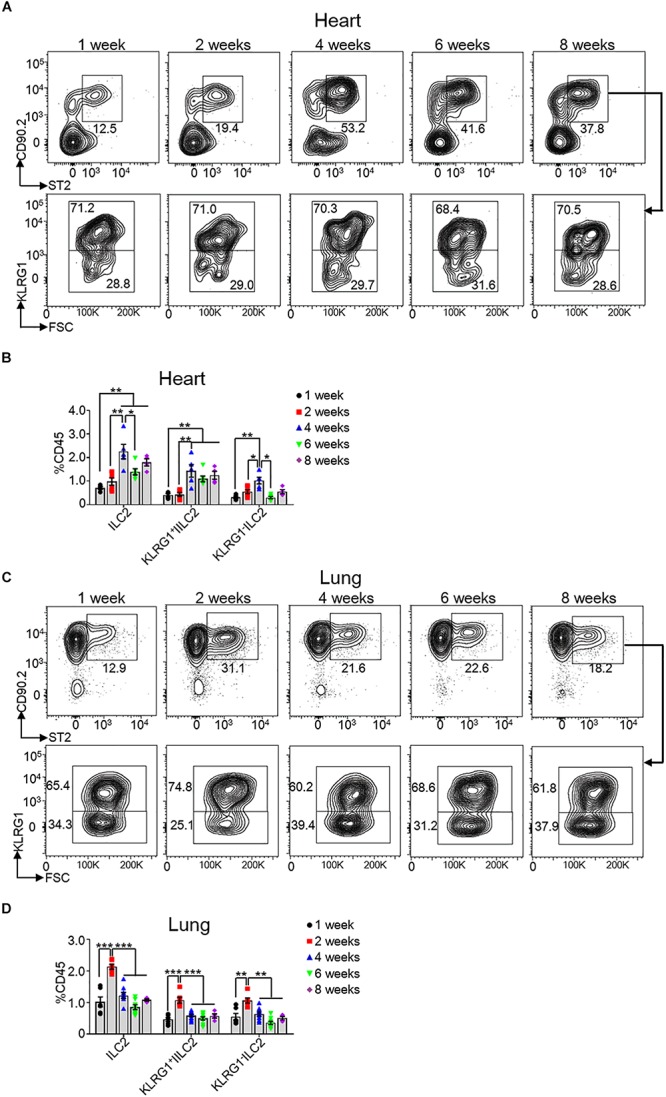
Kinetics of heart ILC2s development after birth. **(A,C)** Flow cytometric analysis of ILC2, KLRG1^+^ ILC2 and KLRG1^–^ ILC2 in the heart **(A)** and lung **(C)**, respectively, of mice at the indicated age after birth. **(B,D)** Cumulative frequencies of ILC2s in the heart **(B)** and in the lung **(D)** of mice at the indicated age after birth. Each dot represents one mouse; error bars represent SEM; *p < 0.05, **p < 0.01, ***p < 0.001. One-way ANOVA **(B,D)**.

All ILCs initially generate in E13.5 fetal liver and seed tissues during fetal development (25). To explore the kinetics of heart ILC2s during development, we determined the ratios of total and each subset of ILC2s at the age of 1, 2, 4, 6, and 8 weeks in both heart and lung tissue. The data revealed that the frequencies of heart total and each subset of ILC2s (including KLRG1^+^ILC2 and KLRG1^–^ILC2) peaked at the age of 4 weeks after birth (Figures 2A,B), while the frequencies of lung ILC2s and subsets peaked at the age of 2 weeks after birth (Figures 2C,D). We also determined the ratio of Type I ILCs, ILC2s and ILC3s in mouse heart at E18.5 and post-birth day 1. The results showed that ILC2s existed, while there were very few type 1 ILCs (including ILC1 and NK cells) and no ILC3s, in mouse heart at both E18.5 and post-birth day 1 (Supplementary Figure S1).

### Heart ILC2s Have Unique Phenotypes Compared With Lung ILC2s

**FIGURE 3:**
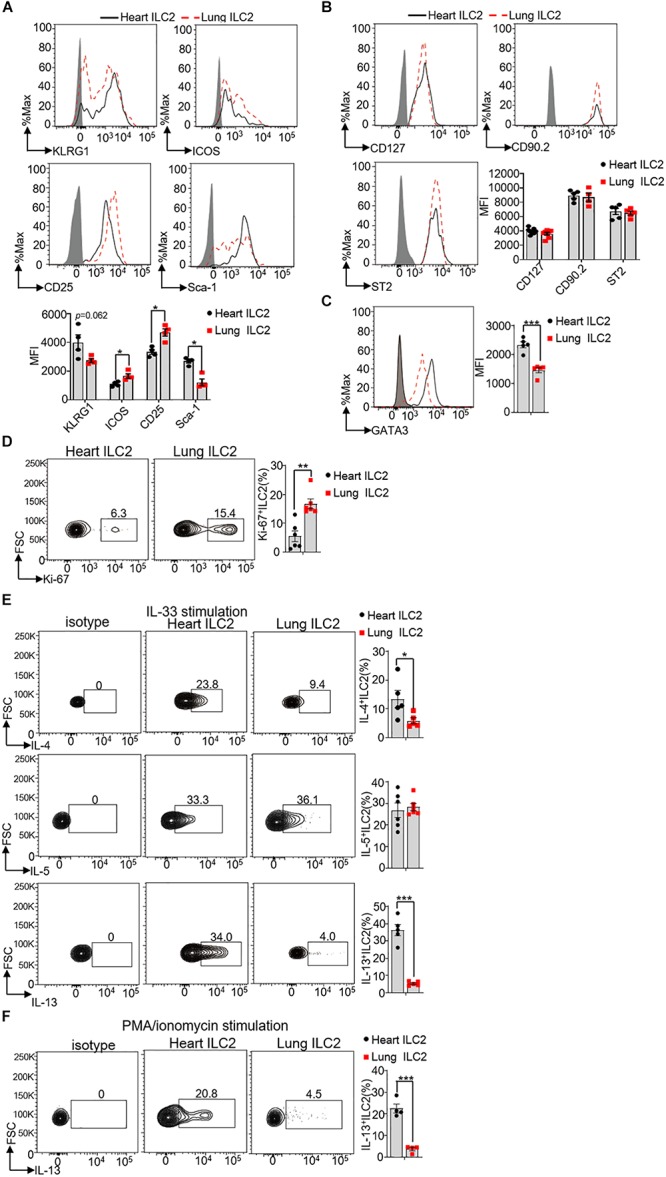
Phenotype differences between heart and lung ILC2s. **(A)** Histograms of cell surface expression and the mean fluorescence intensity (MFI) of KLRG1, ICOS, CD25 and Sca-1 in heart and lung ILC2s (identification as CD45^+^Lin^–^CD127^+^CD90.2^+^ST2^+^cells) of 8 weeks old mice. **(B)** CD127, CD90.2 and ST2 in heart and lung ILC2s (identification as CD45^+^Lin^–^CD127^+^CD25^+^GATA3^+^cells) of 8 weeks old mice. **(C)** The relative expression of GATA3 in heart and lung ILC2s, respectively, of 8 weeks old mice. **(D)** Flow cytometric analysis and cumulative frequencies of Ki-67-expressing ILC2s in heart and lung, respectively, of 8 weeks old mice. **(E)** Flow cytometric analysis and cumulative frequencies of IL-4 (upper), IL-5 (middle) and IL-13 (lower) by heart and lung ILC2s, respectively, following stimulation with IL-33 in the presence of Golgi Plug for 4 h of 8 weeks old mice. **(F)** Flow cytometric analysis and cumulative frequencies of IL-13 by heart and lung ILC2s, respectively, following stimulation with PMA/ionomycin in the presence of Golgi Plug for 4 h of 8 weeks old mice. Each dot represents one mouse; error bars represent SEM; *p < 0.05, **p < 0.01, ***p < 0.001. Unpaired two-tailed Student’s *t*-test **(A–F)**.

Next, we investigated whether heart ILC2s were different from lung ILC2s in terms of surface markers, transcription factor, proliferation and ability of cytokines secretion. Specifically, we gated CD45^+^Lin^–^CD127^+^CD90.2^+^ST2^+^ for ILC2s in the heart and lung to measure the expression levels of KLRG1, ICOS, CD25, Sca-1, GATA3, and Ki-67. Besides, we gated CD45^+^Lin^–^CD25^+^GATA3^+^ for ILC2s to measure the expression levels of CD127, CD90.2, and ST2. Our finding clearly implied that the protein levels of CD127 (IL-7R), CD90.2 (Thy1.2), ST2 (IL-33R), and KLRG1 in heart ILC2s were similar to lung ILC2s, whereas the protein levels of ICOS and CD25 (IL-2Rα) were lower in heart ILC2s than these in lung ILC2s (Figures 3A,B). In contrast, the protein level of Sca-1 in heart ILC2s was higher as compared with that in lung ILC2s (∼2.2-fold). A significant increasing of GATA3 was found in heart ILC2s compared with lung ILC2s (∼1.6-fold) (Figure 3C). As seen in the Figure 3D, heart ILC2s had a weaker proliferation ability than lung ILC2s, indicated by Ki-67 positive cells (∼0.33-fold).

In respond to the cytokines IL-25, TSLP, and IL-33, and ILC2s are the potent sources to produce IL-4, IL-5, and IL-13. Both IL-4 and IL-13 could induce smooth-muscle contraction and wound repairing after infections (26, 27). We therefore stimulated isolated mouse heart and lung lymphocytes with IL-33, following determined the production of IL-4, IL-5, and IL-13 by ILC2s. Compared with lung ILC2s, heart ILC2s had a stronger ability to produce IL-4 and IL-13 (∼2.4-fold and ∼6.8-fold in IL-33 stimulation, respectively) (Figure 3E). Heart ILC2s and lung ILC2s had the similar ability to produce IL-5 (∼0.92-fold) (Figure 3E). Besides, compared with lung ILC2s, heart ILC2s also had a stronger ability to produce IL-4 in response to PMA/ionomycin (Figure 3F). These results suggest that heart ILC2s had unique phenotypes in terms of surface marker, transcription factor, proliferation and cytokine production.

### Circulating Cells Minimally Replace Heart ILC2s

**FIGURE 4:**
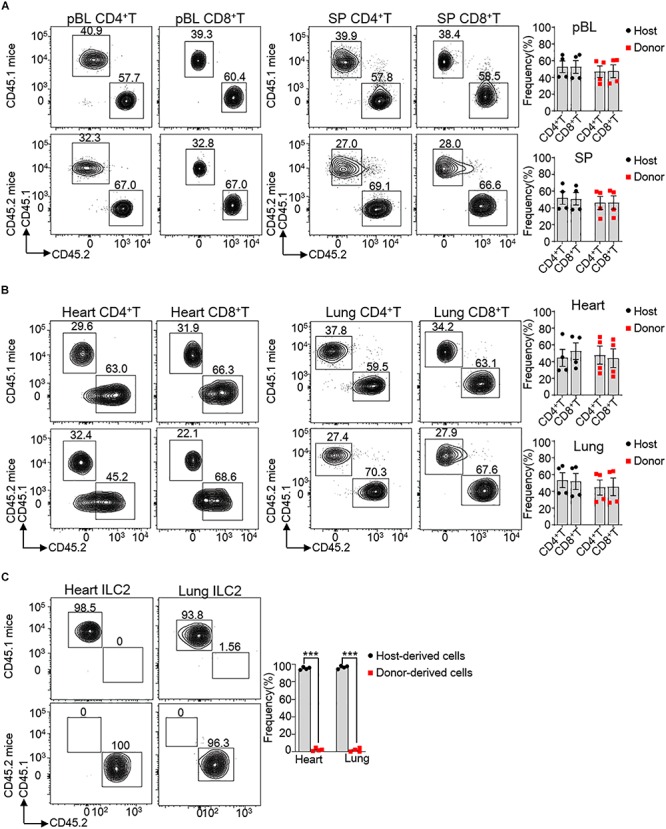
Circulating cells minimally contribute to heart ILC2s renew. **(A,B)** Flow cytometric analysis and cumulative frequencies of CD4^+^ T cells and CD8^+^ T cells in peripheral blood (pBL) and spleen (SP) **(A)** as well as heart and lung **(B)** of parabiotic mice after 2 months of parabiosis between 8 weeks old WT CD45.1 and WT CD45.2 C57BL/6 mice. **(C)** Flow cytometric analysis and cumulative frequencies of ILC2s in heart and lung of parabiotic mice after 2 months of parabiosis between WT CD45.1 and WT CD45.2 C57BL/6 mice. Each dot represents one mouse; error bars represent SEM; ***p < 0.001. Unpaired two-tailed Student’s *t*-test **(A–C)**.

Consideration of affluent bloodstream in the heart, we tested directly whether hematogenous precursors continuously replenished the pool of heart ILC2s in 8 weeks old mice. For this reason, we generated parabiotic mice model, which widely used for the verification of tissue-resident cells in non-lymphoid tissues (13, 28). After 2 months of parabiosis, we analyzed the percentages of various lymphocyte subsets that derived from the donor or host parabiont. Our results clearly show that about 46.8% of CD4^+^ T and 47.4% CD8^+^ T cells in the peripheral blood (pBL) versus about 45.8% of CD4^+^ T and 46.3% CD8^+^ T cells in the spleen (SP) belonged to the parabiont donor (Figure 4A), suggesting that the circulatory system was balanced between the parabiotic mice. Besides, about 47.7% of CD4^+^ T and 44.0% CD8^+^ T cells in the heart tissue and about 44.6% of CD4^+^ T and 45.4% CD8^+^ T cells in the lung tissue derived from parabiont donor, which demonstrated that circulating T cells infiltrated adequately in local tissues (Figure 4B). Remarkably, very few heart ILC2s (∼2.0%) were derived from the blood, the same as lung ILC2s (∼1.1%) (Figure 4C). This indicates that heart ILC2s are initially generated and seed tissues during fetal development and regenerate predominantly through local renewal.

### Heart ILC2s Rapidly Expand and Secrete IL-4 During Myocardial Necroptosis

**FIGURE 5:**
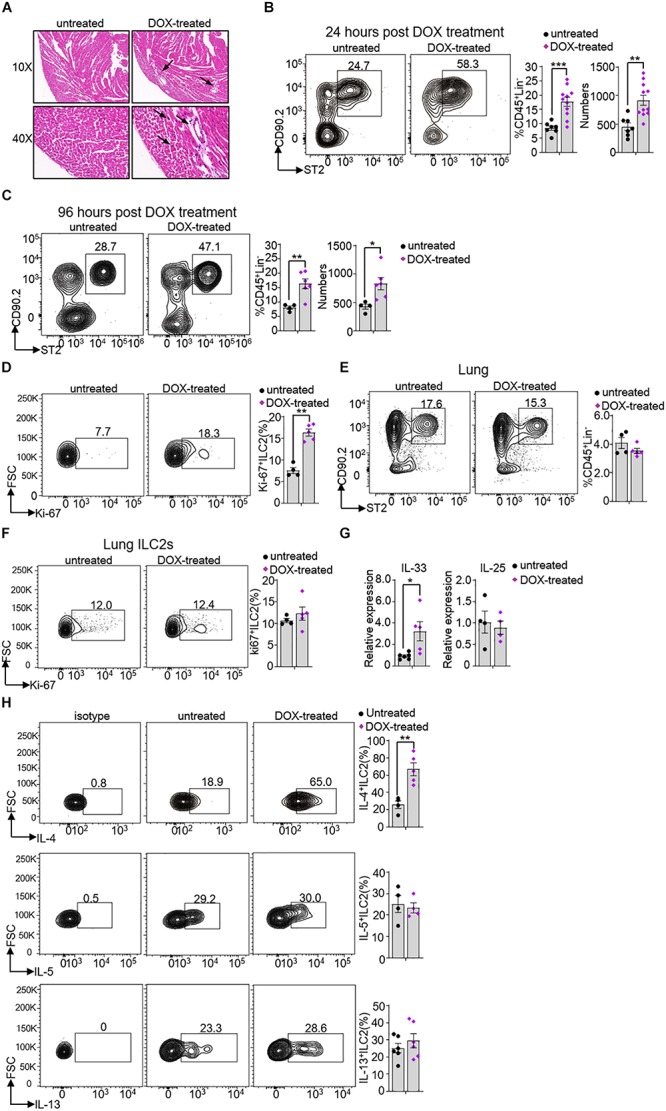
Heart ILC2s expansion and cytokine secretion in response to Doxorubicin treatment. **(A)** Hematoxylin-eosin (HE) staining and representative pictures from heart of 8 weeks old mice after 24 h DOX treatment. The arrow direction indicates representative changes. **(B,C)** Flow cytometric analysis, cumulative frequencies and enumeration of ILC2s in the heart of 8 weeks old mice after 24 h **(B)** and 96 h **(C)** DOX treatment. **(D)** Flow cytometric analysis and cumulative frequency of Ki-67-expressing ILC2s in heart of 8 weeks old mice after 24 h DOX treatment. **(E)** Flow cytometric analysis and cumulative frequency of ILC2s in the lung of 8 weeks old mice after 24 h DOX treatment. **(F)** Flow cytometric analysis and cumulative frequency of Ki67-expressing ILC2s in lung of 8 weeks old mice after 24 h DOX treatment. **(G)** The relative mRNA expression of *IL-33* and *IL-25* in the heart tissue of 8 weeks old mice after 24 h DOX treatment. **(H)** Flow cytometric analysis and cumulative frequencies of IL-4-producing (upper), IL-5-producing (middle) and IL-13-producing ILC2s (lower) in the heart tissue of mice after 24 h DOX treatment, *n* = 4–11. Error bars represent SEM; *p < 0.05, **p < 0.01, ***p < 0.001. Unpaired two-tailed Student’s *t*-test **(B–H)**.

Necroptosis and apoptosis are crucially involved in severe cardiac pathological conditions, including myocardial infarction, ischemia-reperfusion injury and heart failure (14). To investigate whether ILC2s may participate in this process, we established a mouse model of oxidative stress-induced myocardial necroptosis (14). We used the DOX, a well-evaluated chemotherapeutic agent, to establish the irreversible cardiac toxicity, including massive cardiomyocytes loss, cardiomyopathy and heart failure (29, 30). DOX-induced heart injury was firstly confirmed by hematoxylin-eosin (HE) staining. Compared to untreated mice, the DOX-treated mice had significant myocardium necrosis along with nuclear enlargement and swollen of cardiomyocytes (Figure 5A). Meanwhile, we found that the frequency and number of ILC2s were significant higher in DOX-treated mouse (17.68 ± 4.46) than that in untreated mouse (8.51 ± 1.87) after 24 h treatment (Figure 5B) as well as after 96 h treatment (Figure 5C). The frequency of Ki-67^+^ ILC2s was increased in DOX-treated mice (16.5 ± 1.8), compared with that in untreated mice (7.1 ± 1.4) after 24 h treatment (Figure 5D). Interestingly, the frequency and proliferation activity of lung ILC2s were not changed after 24 h DOX treatment (Figures 5E,F). In addition, we also found that the frequencies of total macrophages (CD11b^+^F4/80^+^cells) and type 1 conventional dendritc cells (cDC1s) (CD11b^–^CD11c^+^MHCII^+^), which were involved in heart injury (31, 32), were not noticeable changed after 24 h DOX treatment (Supplementary Figures S2A,B).

Because IL-33 and IL-25 are reported to promote ILC2s proliferation and activation (33, 34), we measured the *IL-33* and *IL-25* mRNA expression in the heart tissue. *IL-33* but not *IL-25* mRNA expression level increased after DOX treatment (∼3.5-fold and ∼0.87-fold, respectively) (Figure 5G). Compared with control mice, heart ILC2s produced more IL-4, but not IL-5 and IL-13 (∼2.6-fold, ∼1.1-fold and ∼1.2-fold, respectively) (Figure 5H). We also measured the CD3^+^T cells and IL-4^+^T cells and both of them were not significant changed after 24 h DOX treatment (Supplementary Figure S2C). Thus, the obtained data proposes that rapid IL-33 production resulted in ILC2s expansion and IL-4 secretion prior to other immune cells during DOX-induced myocardial necroptosis.

## Discussion

In this report, we present the detailed analysis of various subsets of ILCs and phenotypes in the heart tissue. The findings of this study demonstrates the predominant heart ILC2s subset, even greater than lung ILC2s. Our results illustrated that ILC2s at the age of 4 weeks after birth can confidential as heart tissue of mouse. Notably, the heart ILC2s were characterized by lower expression of ICOS, CD25 (IL-2Rα), Ki-67, as well as higher expression of IL-4, IL-13, Sca-1 and GATA3. Our results highlighted that heart-resident ILC2s showed tissue-specific phenotypes and rapidly responded to DOX-induced cardiotoxicity.

Almost all subsets of ILCs and ILC precursors express IL-7R (CD127) and response to IL-7 stimulation (3). We found that CD45^+^Lin^–^CD127^+^ CD90.2^+^ST2^+^ ILC2s, defined in lung tissue (8), was the most dominant ILC subset in mouse heart, while CD45^+^Lin^–^ CD127^+^NK1.1^+^NKp46^+^ Type I ILCs (15), and CD45^+^Lin^–^CD127^+^RORγt^+^ILC3s (22) was merely found in mouse heart. ILC2s were the predominant part of ILCs in human and mouse heart tissue (11), although the authors and we used different gating strategy. The authors identified that ILC2s (identified as CD45^+^Lin^–^CD90^+^RORγt^–^T-bet^–^ST2^+^KLRG1^+^) accounted for about 20% of CD45^+^ Lin^–^CD90^+^ cells in the mice heart tissue, however, our data showed that ILC2s accounted for about 40% of CD45^+^ Lin^–^CD90^+^ CD127^+^ cells. This might because that a part of CD45^+^Lin^–^CD90^+^ cells did not express CD127 (Data not shown).

All ILCs generate and seed tissues during fetal development and the perinatal period is a critical window for the distribution of innate tissue-resident immune cells within developing organs (25, 35). Unlike tissue macrophages, a majority of peripheral ILC2 pools are generated *de novo* during the postnatal window (5, 8), by display little hematogenous redistribution to other tissues (28). Although a minor contribution from circulating precursors can contribute to tissue pools, ILCs regenerate predominantly through local renewal after birth in the resting state (5). Our study also suggested that circulating cells minimally replace heart ILC2s under physiological status. However, whether circulating ILC2s or interorgan migration of tissue-resident ILC2s contributes to heart ILC2s under pathophysiological status are still unknown. This is because a recent study reported that a population of inflammatory ILC2s (iILC2s), which are circulating cells and derived from intestinal ILC2s, could migrate to the lung after IL-25 stimulation or helminth infection (36). Thus, although we found obvious proliferation of ILC2s in heart tissue after DOX treatment, we still could not exclude the possibility that ILC2s migrate to heart from other organs when the heart damage occurs.

During the alveolar phase of lung development, the increasing production of IL-33 accumulates ILC2 cells and the frequency of ILC2s in the mouse lung reached the peak at the age of 2 weeks after birth (8). But, heart resident ILC2s peaks at the age of 4 weeks, which may be due to the lower IL-33 production by cardiac fibroblasts during heart development (37) or less antigens exposure delays the development of ILC2s in the heart (35). In our study, increased IL-33 expression are parallel with increased Ki-67^+^ ILC2s after DOX treatment indicated that IL-33 signal pathway in the heart is important for maintenance of ILC2s. In addition, previous study indicated that IL-4 can activate STAT6 and then induce the expression of GATA3, which forms a positive feedback loop to reinforce Th2 differentiation (38, 39). Thus, we assumed that this IL-4/STAT6/GATA3 axis maybe also take effect in heart ILC2s development.

Compared with lung ILC2s in the lung, heart ILC2s have unique features in the terms of surface marker, such as lower expression level of ICOS and CD25 and higher expression level of Sca-1. ICOS is an important molecule in T cell signal transduction (40) and deficiency of ICOS showed decreased ratio of ILC2s and cytokine production (41, 42). CD25 is a key receptor of IL-2 signaling, which regulates cells survival (43). Besides, Sca-1 is surface molecule stem cell antigen-1, representing the differentiation potential (44). Consistent with these, heart ILC2s showed lower expression of Ki-67. As compared with lung tissue, heart ILC2s might be with a lower proliferation capacity and a more immature phenotype, which might because of the relative sterile micro-environment. Lung ILC2s must maintain a higher proliferative level to expand rapidly in response to various stimulations, such as antigens, virus and worm (8, 45). Interestingly, compared to lung ILC2s (46), heart ILC2s have a stronger ability to produce IL-4 and IL-13 in response to IL-33 or PMA/ionomycin stimulation. Previous study have demonstrated that GATA3 together with STAT6 promotes the expression of IL-4 and IL-13 (47–49). So, these evidence suggest that higher GATA3 expression level of heart ILC2s might be responsible for the higher capacity of IL-4 and IL-13 production. These difference between heart ILC2s and lung ILC2s further demonstrates that the local tissue microenvironment had a profound influence on cells phenotype and function.

Myocardial damage causes sterile inflammation, by recruitment and activation of innate and adaptive immune system cells (31, 50). In this study, we found that ILC2s expanded and produced IL-4 immediately after DOX-induced myocardial necroptosis prior to macrophage, dendrtic cells and IL-4^+^T activation. IL-4 is well-known to regulate a variety of immune responses, including T-cell differentiation and macrophage M2 polarization (51, 52). Previous studies showed that IL-4 serves as an early endogenous neuroprotective mechanism soon after stroke onset and is important in the acute stages of stroke (53, 54). Thus, we speculate that in response to myocardial damage, heart ILC2s act as the first line of responder and produce IL-4 to promote the response during inflammation and cardiac tissue repair. However, production of IL-4 by ILC2s and T cells persistent in the end of recovery stage may also promote myocardial fibrosis (55). In the line with previous study, IL-4 could upregulated the expression of procollagen genes and stimulates collagen production in mouse cardiac fibroblasts (56).

Overall, ILC2s with unique phenotypes are the major subset of ILCs in the heart and different from lung ILC2s in mouse model. Importantly, ILC2s could expand and activate immediately in response to heart damage. Our finding raises the potential for IL-33-elicited ILC2s response as therapeutics for attenuating heart damage.

## Limitation

A tissue-specific knock-out mouse model of ILCs and acquirement enough amount of ILCs to transplant are some significant limitations in the current work. Undoubtedly, future well-accepted studies would be needed to provide the localization of ILC2s within the heart and more direct evidence of a functional requirement for ILC2s in this cardiac injury model.

## Data Availability Statement

All datasets generated for this study are included in the article/Supplementary Material.

## Ethics Statement

All animal experiments were carried out in accordance with the recommendations of the Animal Ethics Committee of the Army Medical University. The animal experiment protocols were approved by the Animal Ethics Committee of the Army Medical University.

## Author Contributions

The work presented was performed in collaboration with all authors. YaD designed and performed the experiments, analyzed the data, and wrote the manuscript. SW, YY, MM, XC, and SC performed the experiments. LL, YG, YC, and SI designed the experiments and edited the manuscript. BC, SL, and XL designed the research and supervised the study. YoD devised the concept, designed the research, supervised the study, and wrote the manuscript.

## Conflict of Interest

The authors declare that the research was conducted in the absence of any commercial or financial relationships that could be construed as a potential conflict of interest.

## Supplementary Material

FIGURE S1 Heart ILC2s exist before birth. Flow cytometric analysis as described in Figure 1A and cumulative frequencies of type I ILCs (including ILC1 and NK cells), ILC2s and ILC3s in the heart of mice at the E18.5 and post-birth day 1. The number inside of gate indicates cell events. Each dot represents one mouse; error bars represent SEM; ^∗∗^*p* < 0.01, ^∗∗∗^*p* < 0.001. One-way ANOVA.

FIGURE S2 Macrophages, dendritic cells (DCs) and IL-4^+^ CD4^+^ T cells in the heart are not increased after 24 h of DOX treatment. **(A,B)** Flow cytometric analysis and cumulative frequencies of macrophages (CD11b^+^F4/80^+^ cells) **(A)** and type 1 conventional dendritc cells (cDC1s) (CD11b^–^CD11c^+^ MHCII^+^ cells) **(B)** in the heart of mice after 24 h DOX treatment. **(C)** Flow cytometric analysis and cumulative frequencies of CD3^+^ T and IL-4^+^ CD4^+^ T cells in the heart following stimulation with PMA/ionomycin in the presence of Golgi Plug for 4 h of mice after 24 h DOX treatment, *n* = 3–4. Error bars represent SEM. Unpaired two-tailed Student’s *t*-test **(A–C)**.

## References

[1] Vivier E Artis D Colonna M Diefenbach A Di Santo JP Eberl G Innate lymphoid cells: 10 years on. *Cell.* (2018) 174:1054–66. 10.1016/j.cell.2018.07.017 30142344

[2] Eberl G Di Santo JP Vivier E. The brave new world of innate lymphoid cells. *Nat Immunol.* (2015) 16:1–5. 10.1038/ni.3059 25521670

[3] Ishizuka IE Constantinides MG Gudjonson H Bendelac A. The innate lymphoid cell precursor. *Ann Rev Immunol.* (2016) 34:299–316. 10.1146/annurev-immunol-041015-055549 27168240

[4] Zook EC Kee BL. Development of innate lymphoid cells. *Nat Immunol.* (2016) 17:775–82. 10.1038/ni.3481 27328007

[5] Kotas ME Locksley RM. Why innate lymphoid cells? *Immunity.* (2018) 48:1081–90. 10.1016/j.immuni.2018.06.002 29924974 PMC6145487

[6] Kim CH Hashimoto-HillM S. Migration and tissue tropism of innate lymphoid cells. *Trends Immunol.* (2016) 37:68–79. 10.1016/j.it.2015.11.003 26708278 PMC4744800

[7] Xue L Salimi M Panse I Mjosberg JM McKenzie AN Spits H Prostaglandin D2 activates group 2 innate lymphoid cells through chemoattractant receptor-homologous molecule expressed on TH2 cells. *J Allergy Clin Immunol.* (2014) 133:1184–94. 10.1016/j.jaci.2013.10.056 24388011 PMC3979107

[8] de Kleer IM Kool M de Bruijn MJ Willart M van Moorleghem J Schuijs MJ Perinatal activation of the interleukin-33 pathway promotes type 2 immunity in the developing lung. *Immunity* (2016) 45:1285–98. 10.1016/j.immuni.2016.10.031 27939673

[9] Eberl GD Littman R. Thymic origin of intestinal alphabeta T cells revealed by fate mapping of RORgammat+ cells. *Science.* (2004) 305:248–51. 10.1126/science.1096472 15247480

[10] Klose CS Flach M Mohle L Rogell L Hoyler T Ebert K Differentiation of type 1 ILCs from a common progenitor to all helper-like innate lymphoid cell lineages. *Cell.* (2014) 157:340–56. 10.1016/j.cell.2014.03.030 24725403

[11] Bracamonte-Baran W Chen G Hou X Talor MV Choi HS Davogustto G Non-cytotoxic cardiac innate lymphoid cells are a resident and quiescent type 2-commited population. *Front Immunol.* (2019) 10:634. 10.3389/fimmu.2019.00634 30984196 PMC6450181

[12] Hashimoto D Chow A Noizat C Teo P Beasley MB Leboeuf M Tissue-resident macrophages self-maintain locally throughout adult life with minimal contribution from circulating monocytes. *Immunity.* (2013) 38:792–804. 10.1016/j.immuni.2013.04.004 23601688 PMC3853406

[13] Kamran P Sereti KI Zhao P Ali SR Weissman IL Ardehali R. Parabiosis in mice: a detailed protocol. *J Vis Exp.* (2013) 80:50556. 10.3791/50556 24145664 PMC3938334

[14] Zhang T Zhang Y Cui M Jin L Wang Y Lv F CaMKII is a RIP3 substrate mediating ischemia- and oxidative stress-induced myocardial necroptosis. *Nat Med.* (2016) 22:175–82. 10.1038/nm.4017 26726877

[15] Wang X Peng H Cong J Wang X Lian Z Wei H Memory formation and long-term maintenance of IL-7Ralpha(+) ILC1s via a lymph node-liver axis. *Nat Commun.* (2018) 9:4854. 10.1038/s41467-018-07405-5 30451860 PMC6242895

[16] Komarowska I Coe D Wang GS Haas R Mauro C Kishore M Hepatocyte growth factor receptor c-met instructs T cell cardiotropism and promotes T cell migration to the heart via autocrine chemokine release. *Immunity.* (2015) 42:1087–99. 10.1016/j.immuni.2015.05.014 26070483 PMC4510150

[17] Guo X Qiu J Tu T Yang X Deng L Anders RA Induction of innate lymphoid cell-derived interleukin-22 by the transcription factor STAT3 mediates protection against intestinal infection. *Immunity.* (2014) 40:25–39. 10.1016/j.immuni.2013.10.021 24412612 PMC3919552

[18] Wang F Meng M Mo B Yang Y Ji Y Huang P Crosstalks between mTORC1 and mTORC2 variagate cytokine signaling to control NK maturation and effector function. *Nat Commun.* (2018) 9:4874. 10.1038/s41467-018-07277-9 30451838 PMC6242843

[19] Huang P Wang F Yang Y Lai W Meng M Wu S Hematopoietic-specific deletion of foxo1 promotes NK cell specification and proliferation. *Front Immunol.* (2019) 10:1016. 10.3389/fimmu.2019.01016 31139183 PMC6519137

[20] Deng Y Kerdiles Y Chu J Yuan S Wang Y Chen X Transcription factor Foxo1 is a negative regulator of natural killer cell maturation and function. *Immunity.* (2015) 42:457–70. 10.1016/j.immuni.2015.02.006 25769609 PMC4400836

[21] Li HB Tong J Zhu S Batista PJ Duffy EE Zhao J m(6)A mRNA methylation controls T cell homeostasis by targeting the IL-7/STAT5/SOCS pathways. *Nature.* (2017) 548:338–42. 10.1038/nature23450 28792938 PMC5729908

[22] Xia P Liu J Wang S Ye B Du Y Xiong Z WASH maintains NKp46(+) ILC3 cells by promoting AHR expression. *Nat Commun.* (2017) 8:15685. 10.1038/ncomms15685 28589939 PMC5467242

[23] Jiao Y Huntington ND Belz GT Seillet C. Type 1 innate lymphoid cell biology: lessons learnt from natural killer cells. *Front Immunol.* (2016) 7:426. 10.3389/fimmu.2016.00426 27785129 PMC5059362

[24] Diefenbach A Colonna M Koyasu S. Development, differentiation, and diversity of innate lymphoid cells. *Immunity.* (2014) 41:354–65. 10.1016/j.immuni.2014.09.005 25238093 PMC4171710

[25] Eberl G Colonna M SantoA J.P. Di McKenzie N. Innate lymphoid cells. Innate lymphoid cells: a new paradigm in immunology. *Science.* (2015) 348:aaa6566. 10.1126/science.aaa6566 25999512 PMC5658207

[26] Artis D Spits H. The biology of innate lymphoid cells. *Nature.* (2015) 517:293–301. 10.1038/nature14189 25592534

[27] Pulendran B Artis D. New paradigms in type 2 immunity. *Science.* (2012) 337:431–5. 10.1126/science.1221064 22837519 PMC4078898

[28] Gasteiger G Fan XY Dikiy S Lee SY Rudensky AY. Tissue residency of innate lymphoid cells in lymphoid and nonlymphoid organs. *Science.* (2015) 350:981–5. 10.1126/science.aac9593 26472762 PMC4720139

[29] Xia P Liu Y Chen J Coates S Liu DX Cheng Z. Inhibition of cyclin-dependent kinase 2 protects against doxorubicin-induced cardiomyocyte apoptosis and cardiomyopathy. *J Biol Chem.* (2018) 293:19672–85. 10.1074/jbc.RA118.004673 30361442 PMC6314117

[30] Abdullah CS Alam S Aishwarya R Miriyala S Bhuiyan MAN Panchatcharam M Doxorubicin-induced cardiomyopathy associated with inhibition of autophagic degradation process and defects in mitochondrial respiration. *Sci Rep.* (2019) 9:2002. 10.1038/s41598-018-37862-3 30765730 PMC6376057

[31] Yan X Anzai A Katsumata Y Matsuhashi T Ito K Endo J Temporal dynamics of cardiac immune cell accumulation following acute myocardial infarction. *J Mol Cell Cardiol.* (2013) 62:24–35. 10.1016/j.yjmcc.2013.04.023 23644221

[32] Swirski FK Nahrendorf M. Leukocyte behavior in atherosclerosis, myocardial infarction, and heart failure. *Science.* (2013) 339:161–6. 10.1126/science.1230719 23307733 PMC3891792

[33] Bartemes KR Iijima K Kobayashi T Kephart GM McKenzie AN Kita H. IL-33-responsive lineage- CD25+ CD44(hi) lymphoid cells mediate innate type 2 immunity and allergic inflammation in the lungs. *J Immunol.* (2012) 188:1503–13. 10.4049/jimmunol.1102832 22198948 PMC3262877

[34] Huang YF Guo LY Qiu J Chen X Hu-Li J Siebenlist U IL-25-responsive, lineage-negative KLRG1(hi) cells are multipotential ‘inflammatory’ type 2 innate lymphoid cells. *Nat Immunol.* (2015) 16:161. 10.1038/ni.3078 25531830 PMC4297567

[35] Schneider C Lee J Koga S Ricardo-Gonzalez RR Nussbaum JC Smith LK Tissue-resident group 2 innate lymphoid cells differentiate by layered ontogeny and in situ perinatal priming. *Immunity.* (2019) 50:1425–1438e5. 10.1016/j.immuni.2019.04.019 31128962 PMC6645687

[36] Huang Y Mao K Chen X Sun MA Kawabe T Li W S1P-dependent interorgan trafficking of group 2 innate lymphoid cells supports host defense. *Science.* (2018) 359:114–9. 10.1126/science.aam5809 29302015 PMC6956613

[37] Chen G Bracamonte-Baran W Diny NL Hou X Talor MV Fu K Sca-1(+) cardiac fibroblasts promote development of heart failure. *Eur J Immunol.* (2018) 48:1522–38. 10.1002/eji.201847583 29953616 PMC6696927

[38] Ho IC Miaw SC. Regulation of IL-4 expression in immunity and diseases. *Adv Exp Med Biol.* (2016) 941:31–77. 10.1007/978-94-024-0921-5_3 27734408

[39] Ranganath S Ouyang W Bhattarcharya D Sha WC Grupe A Peltz G GATA-3-dependent enhancer activity in IL-4 gene regulation. *J Immunol.* (1998) 161:3822–6. 9780146

[40] Yoshinaga SK Whoriskey JS Khare SD Sarmiento U Guo J Horan T T-cell co-stimulation through B7RP-1 and ICOS. *Nature.* (1999) 402:827–32. 10.1038/45582 10617205

[41] Paclik D Stehle C Lahmann A Hutloff A Romagnani C. ICOS regulates the pool of group 2 innate lymphoid cells under homeostatic and inflammatory conditions in mice. *Eur J Immunol.* (2015) 45:2766–72. 10.1002/eji.201545635 26249010

[42] Maazi H Patel N Sankaranarayanan I Suzuki Y Rigas D Soroosh P ICOS:ICOS-ligand interaction is required for type 2 innate lymphoid cell function, homeostasis, and induction of airway hyperreactivity. *Immunity.* (2015) 42:538–51. 10.1016/j.immuni.2015.02.007 25769613 PMC4366271

[43] Waters RS Perry JSA Han S Bielekova B Gedeon T. The effects of interleukin-2 on immune response regulation. *Math Med Biol.* (2018) 35:79–119. 10.1093/imammb/dqw021 28339682 PMC5576036

[44] Morcos MNF Schoedel KB Hoppe A Behrendt R Basak O Clevers HC SCA-1 expression level identifies quiescent hematopoietic stem and progenitor cells. *Stem Cell Rep.* (2017) 8:1472–8. 10.1016/j.stemcr.2017.04.012 28506535 PMC5469944

[45] Wojno ED Monticelli LA Tran SV Alenghat T Osborne LC Thome JJ The prostaglandin D(2) receptor CRTH2 regulates accumulation of group 2 innate lymphoid cells in the inflamed lung. *Mucosal Immunol.* (2015) 8:1313–23. 10.1038/mi.2015.21 25850654 PMC4598246

[46] Halim TY Krauss RH Sun AC Takei F. Lung natural helper cells are a critical source of Th2 cell-type cytokines in protease allergen-induced airway inflammation. *Immunity.* (2012) 36:451–63. 10.1016/j.immuni.2011.12.020 22425247

[47] Ouyang W Lohning M Gao Z Assenmacher M Ranganath S Radbruch A Stat6-independent GATA-3 autoactivation directs IL-4-independent Th2 development and commitment. *Immunity.* (2000) 12:27–37. 10.1016/s1074-7613(00)80156-9 10661403

[48] Spilianakis CG Flavell RA. Long-range intrachromosomal interactions in the T helper type 2 cytokine locus. *Nat Immunol.* (2004) 5:1017–27. 10.1038/ni1115 15378057

[49] Lee HJ Takemoto N Kurata H Kamogawa Y Miyatake S O’Garra A GATA-3 induces T helper cell type 2 (Th2) cytokine expression and chromatin remodeling in committed Th1 cells. *J Exp Med.* (2000) 192:105–15. 10.1084/jem.192.1.105 10880531 PMC1887713

[50] Vdovenko D Eriksson U. Regulatory Role of CD4(+) T Cells in Myocarditis. *J Immunol Res.* (2018) 2018:4396351. 10.1155/2018/4396351 30035131 PMC6032977

[51] Liu X Liu J Zhao S Zhang H Cai W Cai M Interleukin-4 is essential for microglia/macrophage M2 polarization and long-term recovery after cerebral ischemia. *Stroke.* (2016) 47:498–504. 10.1161/strokeaha.115.012079 26732561 PMC4729613

[52] Shintani Y Ito T Fields L Shiraishi M Ichihara Y Sato N IL-4 as a repurposed biological drug for myocardial infarction through augmentation of reparative cardiac macrophages: proof-of-concept data in mice. *Sci Rep.* (2017) 7:6877. 10.1038/s41598-017-07328-z 28761077 PMC5537273

[53] Kim HM Shin HY Jeong HJ An HJ Kim NS Chae HJ Reduced IL-2 but elevated IL-4, IL-6, and IgE serum levels in patients with cerebral infarction during the acute stage. *J Mol Neurosci.* (2000) 14:191–6. 10.1385/JMN:14:3:191 10984195

[54] Xiong X Barreto GE Xu L Ouyang YB Xie X Giffard RG. Increased brain injury and worsened neurological outcome in interleukin-4 knockout mice after transient focal cerebral ischemia. *Stroke.* (2011) 42:2026–32. 10.1161/STROKEAHA.110.593772 21597016 PMC3128567

[55] Kanellakis P Ditiatkovski M Kostolias G Bobik A. A pro-fibrotic role for interleukin-4 in cardiac pressure overload. *Cardiovasc Res.* (2012) 95:77–85. 10.1093/cvr/cvs142 22492684

[56] Peng H Sarwar Z Yang XP Peterson EL Xu J Janic B Profibrotic Role for Interleukin-4 in Cardiac Remodeling and Dysfunction. *Hypertension.* (2015) 66:582–9. 10.1161/hypertensionaha.115.05627 26195478 PMC4685692

