# Supplementary figures and images for: Unique Phenotypes of Heart Resident Type 2 Innate Lymphoid Cells
Source: Front Immunol. 2020 May 5;11:802. doi: 10.3389/fimmu.2020.00802 (PMC7214751; doi:10.3389/fimmu.2020.00802)

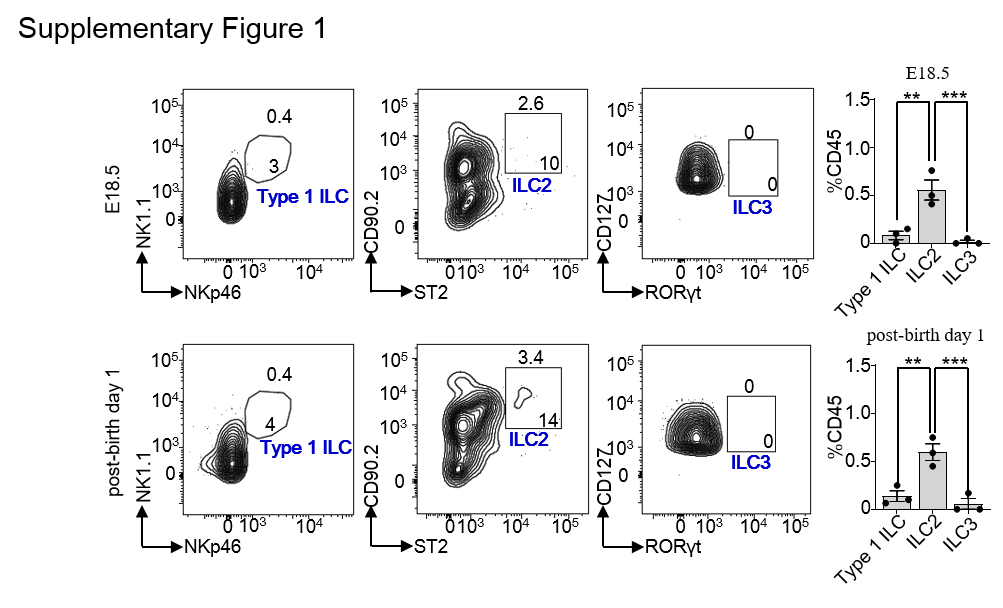

Supplement: FIGURE S1 — Heart ILC2s exist before birth. Flow cytometric analysis as described in Figure 1A and cumulative frequencies of type I ILCs (including ILC1 and NK cells), ILC2s and ILC3s in the heart of mice at the E18.5 and post-birth day 1. The number inside of gate indicates cell events. Each dot represents one mouse; error bars represent SEM; ∗∗p < 0.01, ∗∗∗p < 0.001. One-way ANOVA. [file Image_1.TIF]

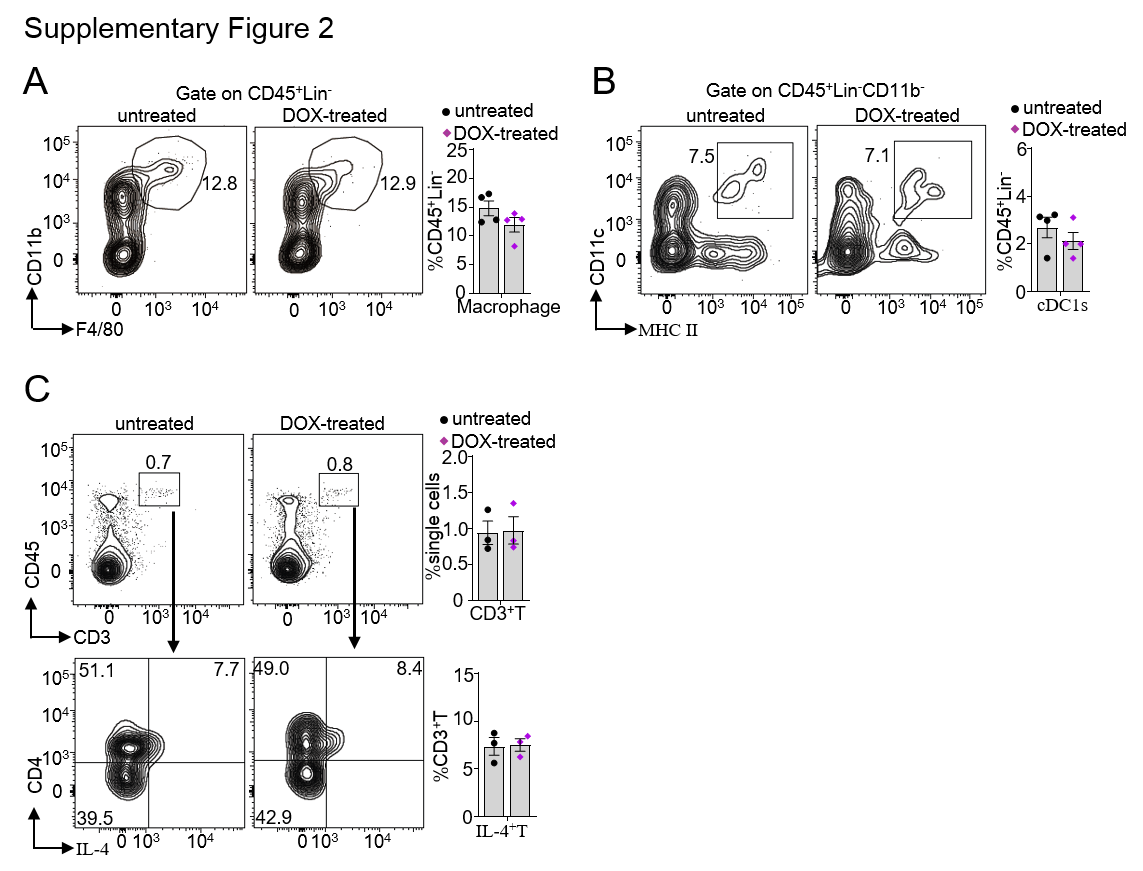

Supplement: FIGURE S2 — Macrophages, dendritic cells (DCs) and IL-4+ CD4+ T cells in the heart are not increased after 24 h of DOX treatment. (A,B) Flow cytometric analysis and cumulative frequencies of macrophages (CD11b+F4/80+ cells) (A) and type 1 conventional dendritc cells (cDC1s) (CD11b–CD11c+ MHCII+ cells) (B) in the heart of mice after 24 h DOX treatment. (C) Flow cytometric analysis and cumulative frequencies of CD3+ T and IL-4+ CD4+ T cells in the heart following stimulation with PMA/ionomycin in the presence of Golgi Plug for 4 h of mice after 24 h DOX treatment, n = 3–4. Error bars represent SEM. Unpaired two-tailed Student’s t-test (A–C). [file Image_2.TIF]
